# Antioxidant Maillard Reaction Products from Milk Whey: A Food By-Product Valorisation

**DOI:** 10.3390/foods14030450

**Published:** 2025-01-30

**Authors:** Sara Bolchini, Tiziana Nardin, Ksenia Morozova, Matteo Scampicchio, Roberto Larcher

**Affiliations:** 1 Faculty of Agricultural, Environmental and Food Science, Free University of Bolzano, 39100 Bolzano, Italy; sbolchini@unibz.it (S.B.); ksenia.morozova@unibz.it (K.M.); matteo.scampicchio@unibz.it (M.S.); 2 Centro di Trasferimento Tecnologico, Fondazione Edmund Mach, 38010 San Michele all’Adige, Italy; roberto.larcher@fmach.it

**Keywords:** Maillard reaction, food by-product valorisation, antioxidants, milk whey, high-resolution mass spectrometry

## Abstract

The Maillard reaction (MR) is a key process in food science, producing bioactive compounds with antioxidant properties. This study evaluates the antioxidant potential of MR products (MRPs) from different dairy byproducts—cow cheese whey, goat cheese whey, and cow yoghurt whey—highlighting their applicability in food preservation and waste valorisation. Whey samples were subjected to the MR at 140 °C for 90 min, showing significant amino acid and sugar consumption, particularly arginine, histidine, and lactose. Using a library of potential antioxidant MRPs (molecular weight < 250 Da), 28 key compounds, including 2-pyrrolecarboxaldehyde and maltol isomer, were identified, primarily in cow cheese whey. A complementary high-molecular-weight MRP library (≥250 Da) identified 72 additional antioxidant compounds, with distinct production patterns linked to whey type. Multivariate analyses confirmed that whey type strongly influences MRP profiles. These results highlight the potential of MR to transform whey by-products into valuable sources of natural antioxidants. This approach offers sustainable strategies for enhancing food preservation, reducing food waste, and supporting the targeted use of MRPs in the food industry.

## 1. Introduction

### 1.1. Milk Whey

Milk whey is the liquid by-product formed after curdling and straining milk during cheese or yoghurt production [1,2]. It is a nutrient-rich source of proteins, lactose, vitamins, and minerals, making it a versatile component in food and nutritional products. Whey is especially abundant in high-quality proteins, such as β-lactoglobulin and α-lactalbumin, which are easily digestible and offer several health benefits [3].

There are two main types of milk whey, derived from two different productions: cheese whey and yoghurt whey. There are notable compositional differences between cheese whey, and yoghurt whey. Cheese whey, also known as sweet whey, is generated when milk is coagulated with enzymes like rennet during cheese-making. It has lower acidity and retains more lactose. In contrast, yoghurt whey is produced during the fermentation of yoghurt, where bacteria convert lactose into lactic acid, giving it higher acidity and a slightly altered protein profile. Yoghurt whey typically contains more lactic acid and probiotics, enhancing its nutritional properties. Moreover, milk whey composition is strictly correlated with the animal that produces the milk (cow, goat, sheep, etc.), varying in the type and quantity of molecules present [4]. The valorisation of milk whey is crucial as it transforms a widely available by-product, that otherwise would be discarded, into valuable resources, reducing waste and promoting circular economy principles. Additionally, it could enable the production of natural food additives like preservatives, offering eco-friendly alternatives to synthetic compounds and supporting clean label trends in the food industry [5].

### 1.2. Food Preservation

Food preservation extends the shelf life of perishable products by slowing down spoilage. Traditional methods such as drying, salting and fermentation have been used for centuries. In modern times, chemical preservatives are added to maintain freshness [6]. Growing concern about the health risks associated with synthetic additives has led to interest in natural food preservatives [7].

Natural preservatives maintain food safety and quality, and meet consumer demand for healthier, clean-label products [8,9,10].

#### Antioxidants

Within the large class of food preservatives, antioxidants play a key role in maintaining food quality and safety. Antioxidants protect food from oxidation, which causes spoilage, rancidity and the loss of nutritional value [11]. They neutralise free radicals, which contribute to oxidation. Common antioxidants used as food preservatives include vitamin C, vitamin E and plant-based polyphenols [12].

These substances help to extend the shelf life of food while preserving its taste, appearance and nutritional quality. They are essential for maintaining the freshness of products such as oils, meats and processed foods [13].

### 1.3. Food Waste Valorisation

Food waste valorisation is the process of converting discarded food or food by-products into valuable products, turning what would otherwise be waste into resources for other industries. This process not only helps to reduce food waste, but also creates economic value by producing biofuels, animal feed, fertilisers and even ingredients for cosmetics or pharmaceuticals and food additives like food preservatives. By reusing by-products like milk whey, food waste valorisation supports sustainability efforts, reduces environmental impact and contributes to a circular economy where resources are efficiently reused rather than discarded [5].

The growing interest in food by-product valorisation has many causes. Food waste in landfills produces harmful greenhouse gases, contributing to climate change. Valorisation reduces waste, turning by-products into products. Food security is also a key aspect to be considered. Reducing waste makes better use of food resources, which is vital in the context of population growth and food shortages. Increased consumer awareness and stricter government regulations have driven industries to seek innovative solutions to valorise food waste. Food waste is a significant global problem and finding ways to valorise waste can contribute to sustainability goals [14]. Particularly, milk whey disposal poses significant financial and environmental challenges. Financially, dairy producers incur high treatment and disposal costs due to whey’s organic content and limited reuse options. Environmentally, improper disposal can lead to pollution as whey’s high biological oxygen demand (BOD) depletes the oxygen in water, harming aquatic ecosystems and contributing to eutrophication [15]. Addressing these issues requires investment in sustainable whey management strategies. By treating food waste like milk whey with simple and naturally occurring processes, like the Maillard reaction, it could be possible to extract value from otherwise discarded materials [16,17].

### 1.4. Maillard Reaction

The Maillard reaction is a complex chemical process that occurs between amino acids and reducing sugars when exposed to heat, resulting in the browning of food and the development of rich, complex flavours [18,19]. Discovered by French chemist Louis Camille Maillard in 1912, this reaction is a cornerstone of culinary science and is responsible for the appealing taste, aroma and colour of a wide range of foods, including roasted coffee, grilled meats, baked bread and fried potatoes. It plays a crucial role in cooking and food science by enhancing flavour profiles and contributing to the sensory experience of eating [20,21]. The Maillard reaction also has implications in areas beyond cooking, including food preservation and nutrition [22,23].

#### 1.4.1. Antioxidant Activity of MRPs

As well as contributing to the flavour, aroma and colour of foods, the Maillard reaction also produces compounds known for their antioxidant and antimicrobial activity [19,24,25,26]. When food undergoes the Maillard reaction during thermal processing, various products are formed, including melanoidins and other intermediates, which have been shown to have significant antioxidant properties. These Maillard reaction products (MRPs) can scavenge free radicals, inhibit lipid oxidation and reduce oxidative stress in both food systems and biological processes [8].

The antioxidant activity of MRPs is of growing interest in food science and health, as oxidative stress has been linked to food spoilage, quality degradation and several chronic human diseases [23,27]. Incorporating MRPs into foods can improve shelf life and potentially enhance nutritional benefits. However, the extent of their antioxidant capacity can vary depending on factors such as the specific sugars and amino acids involved, temperature, pH and the duration of heating [28]. Therefore, understanding the antioxidant potential of MRPs is key to both food preservation and the promotion of health through diet. Indeed, antioxidant compounds from the MR, like melanoidins, can help neutralise free radicals, reduce oxidative stress, and lower the risk of chronic diseases such as heart disease and diabetes. They also have anti-inflammatory and antimicrobial benefits, supporting overall health [29].

#### 1.4.2. Influence of Variables on MRP Production

The formation of antioxidant MRPs is significantly influenced by reaction conditions, including temperature, time, pH and the nature of the reactants involved. Higher temperatures and longer heating times generally accelerate the Maillard reaction and promote the formation of more complex MRPs with enhanced antioxidant properties [30]. pH also plays a critical role, with alkaline conditions favouring the development of antioxidant compounds [31]. In addition, the specific types of amino acids and reducing sugars involved can affect the yield and potency of antioxidant MRPs, as different combinations result in different reaction pathways and end products [32,33]. By carefully controlling these variables, the antioxidant capacity of MRPs in food processing can be optimised.

### 1.5. Research Gap and Aim of the Work

Even though it is known that the MR produces antioxidant molecules starting from common reactants present in food and its by-products, like milk whey, such as reducing sugars and amino compounds, very little information regarding the optimisation of the process and its application in the food industry are available. Therefore, this work aims to study the development of these naturally produced antioxidant compounds, applying the MR to milk whey, a food chain by-product. The MR was induced on different samples of milk whey, derived from different cheese and yoghurt productions. Moreover, based on the information presented in a previous work [34], the samples were analysed through high-performance liquid chromatography (HPLC) coupled with high-resolution mass spectrometry (HRMS) to detect, select and identify the antioxidants produced through the MR.

## 2. Materials and Methods

### 2.1. Sample Collection

The milk whey (MW) was obtained from local dairy producers. Three different types of whey were used for this study: a cow cheese whey, a goat cheese whey and a cow yoghurt whey. Four 500 mL samples of each type of whey were collected from different production batches over four different days. They were stored at −20 °C for a maximum of 1 month before use.

### 2.2. Samples Characterisation

The pH of all the samples was measured before and after the MR since it is a key variable in the reaction [35]. Moreover, sugar and free amino acid contents were evaluated.

#### 2.2.1. Amino Acid Content

Amino acids (alanine (ala), arginine (arg), asparagine (asn), aspartic acid (asp), citrulline (cit), ethanolamine (MEA), γ butirric acid (gaba), gln (glutamine), glutamic acid (glu), glycine (gly), histidine (hys), isoleucine (ile), leucine (leu), lysine (lys), ornithine (orn), phenylalanine (phe), serine (ser), threonine (thr), tryptophan and methionine (trp + met), tyrosine (tyr) and valine (val) were quantified by high-performance liquid chromatography (HPLC) coupled to a fluorescence detector (FLD) after derivatization with ortho-phthaldehyde (OPA) according to the method described by Gallo et al., 2023 [36]. The analysis was performed on an Agilent 1260 Infinity HPLC system (Agilent Technologies, Santa Clara, CA, USA) equipped with a fluorescence detector set at an excitation wavelength of 336 nm and an emission wavelength of 445 nm. Separation was performed on a Chromolith Performance RP-18e column (100 × 4.6 mm) (Merck, Darmstadt, Germany) with a Chromolith RP-18e guard cartridge (10 × 4.6 mm) (Merck, Darmstadt, Germany), maintained at 40 °C. The mobile phase consisted of 0.05 M of sodium acetate (pH 6.9) as eluent A and methanol as eluent B. The flow rate was maintained at 2 mL/min and the gradient for eluent B was programmed as follows: 0% from 0 to 1 min, 20% from 1 to 11 min, 40% from 11 to 16 min, 100% from 16 to 25 min, 10% from 25 to 27 min and 0% from 27 to 30 min. Samples (10 µL) were diluted (1:5) with MilliQ water, adjusted to a pH of 7, filtered with 0.2 nm polytetrafluoroethylene (PTFE) filters and stored in the autosampler at 10 °C. The instrument automatically performed derivatization by mixing 10 µL of sample with 10 µL of OPA derivatization solution, followed by 1 min of mixing before injection. The derivatizing solution consisted of 4.5 g/L OPA (Sigma-Aldrich, St. Louis, MO, USA) in 0.1 M sodium tetraborate (pH 10.5), 10% methanol and 2% 2-mercaptoethanol (Sigma-Aldrich, St. Louis, MO, USA). Data acquisition and processing were performed using OpenLab CDS 3.1 software (Agilent Technologies, Santa Clara, CA, USA). Amino acid quantification was based on external calibration curves using amino acid standards, with β-glutamic acid as an internal control.

#### 2.2.2. Sugar Content

The chromatographic separation and quantification of sugars followed the method described by Di Lella et al., 2019 [37]. The analysis was performed on an ICS 5000 ion chromatograph (Dionex, Thermo Fisher Scientific, Waltham, MA, USA) equipped with an eluent generator, autosampler, quaternary gradient pump, column oven and pulsed amperometry detector (PAD). The PAD consisted of a gold working electrode and a palladium reference electrode. For the separation of sugars (including monosaccharides and disaccharides), 5 µL of sample was injected into a CarboPac PA200 analytical column (3 × 250 mm), preceded by a CarboPac PA200 guard column (3 × 50 mm), both (Dionex, Thermo Fisher Scientific, Waltham, MA, USA). The column stationary phase was a hydrophobic polymeric pellicular resin with quaternary ammonium as the anion exchange resin functional group. Both columns were maintained at a constant temperature of 30 °C. The flow rate was set at 0.4 mL/min, controlled by an eluent generator which automatically prepared the potassium hydroxide (KOH) eluent by the electrolysis of deionized water. The elution programme consisted of an isocratic elution with 0.1 mM of KOH from 0 to 18 min, followed by a gradient elution from 0.1 to 100 mM of KOH between 18 and 21.5 min, maintained until 27.5 min. The KOH concentration was then reduced back to 0.1 mM to allow the column to equilibrate for 5 min. Deionized water was continuously flushed with helium to prevent the formation of carbonates. Sugar detection was performed using the PAD, with the working pulse potential applied according to a quaternary curve (as shown in Table 1), with reference to the palladium electrode.

### 2.3. Maillard Reaction

All the samples studied were heated to induce a MR. Particularly, 5 mL of each sample were put in a 10 mL Pyrex flask sealed with a PTFE hermetic plug and heated in an oven set at 140 °C for 90 min. The PTFE hermetic plug ensured minimal evaporation and allowed pressure buildup, enabling the sample temperature to rise beyond the boiling point of water. The temperature has been chosen because it is sufficient to guarantee the active form of the sugars, with an open chain, and this allows a faster reaction. Moreover, this temperature allows for reaching the activation energy of almost all the Maillard kinetic steps, guaranteeing that all reactants enter in the MR [38]. The time of reaction was chosen after preliminary experiments indicating that 90 min was enough to achieve a plateau in antioxidant molecule production, monitored through HPLC-HRMS (method the described in Section 2.5.1). The samples, then, were cooled in an ice bath and kept at −20 °C prior to analyses for a maximum of one week.

### 2.4. Isolation, and Peptides’ and Proteins’ Role Evaluation

To evaluate the role of peptides and proteins in the formation of antioxidant MRPs, two samples of the four available for each type of whey were ultrafiltered using Vivaspin^®^ Turbo 15, 3000 MWCO plyethersulfone (PES) centrifugal concentrator filters (Sartorius, Göttingen, Germany). The 3 kDa filters were selected because they allow for the retention of large peptides and proteins while allowing smaller peptides and free amino acids with molecular weights below 3 kDa to pass through. This size cut-off ensures a clear separation of smaller components from larger ones. In particular, 10 mL of each sample were filtered and the retained part was then re-suspended in a 10 mL water solution containing the three main sugars present in milk whey—galactose, glucose and lactose—with concentrations of 38 g/L, 15 g/L and 40 g/L, respectively. These concentrations correspond to the average of the sugar’s concentrations measured in all the samples collected. In the samples obtained, then, the MR was induced as reported in the previous section.

### 2.5. HRMS Antioxidant MRP Analyses

Whey samples were analysed before and after the MR to detect and qualify the antioxidant MRPs produced.

#### 2.5.1. HPLC and MS Method

Samples were analysed on a Dionex UltiMate 3000 HPLC system (Thermo Fisher Scientific, Waltham, MA, USA) equipped with two binary pumps and a temperature-controlled autosampler. Detection was performed using a Q-Exactive Orbitrap high-resolution mass spectrometer (HRMS, Thermo Fisher Scientific, Waltham, MA, USA) with a heated electrospray ionisation (HESI) source operating in both positive and negative ionisation modes. The capillary voltage was set at 2.50 kV and the capillary temperature was maintained at 330 °C. Full MS scans were acquired over a mass range of 50 to 750 *m*/*z* or 200 to 2000 *m*/*z* with a resolution of 70,000 full width at half maximum (FWHM) at 200 *m*/*z*, an automatic gain control (AGC) target of 3 × 10^6^ and a maximum injection time of 100 ms. Data-dependent MS/MS (MS2) analysis was performed to obtain fragmentation patterns for both targeted and untargeted species with an AGC target of 1 × 10^5^, a maximum injection time of 50 ms, a resolution of 17,500 FWHM and an isolation window of 4.0 *m*/*z*. The mass spectrometer was calibrated prior to analysis using Pierce LTQ Velos ESI positive and negative calibration solutions (Thermo Fisher Scientific, Waltham, MA, USA). Data acquisition and analysis were performed using Chromeleon 7.3, Compound Discoverer 3.3.3.200 and Mass Frontier 8.3 software (Thermo Fisher Scientific, Waltham, MA, USA). All the analyses were run using a Dionex IonPac NS2 (4 × 150 mm, 5 µm particle size Thermo Fisher Scientific, Waltham, MA, USA) column. The column temperature was kept constant at 30 °C. The mobile phases used were Milli-Q water with 0.5% formic acid (*v*/*v*) (A) and acetonitrile with 0.5% formic acid (*v*/*v*) (B). Before injection, the samples were diluted 1:10 with MilliQ water, filtered with 0.2 µm PTFE filters and the injection volume was set at 2 µL. The analysis was conducted at a constant flow rate of 0.35 mL/min using the following gradient for optimal chromatographic separation: from 0 to 1 min, 5% of eluent B; from 1 to 10 min, 15% eluent B; from 10 to 15 min, 35% eluent B; and from 15 to 21 min, 5% eluent B.

#### 2.5.2. Study of High-Molecular-Weight Antioxidant MRPs

The MR is a very complex reaction, and MRPs are a wide group of molecules with different bioactivities and different molecular weights. The low-molecular-weight (<250 Da) antioxidant MRPs were already evaluated in a previous work [34] but the potential antioxidant MRPs with higher molecular weights were never considered. Because of these, the same untargeted approach applied in the previous work was used to monitor the production of MRPs with a molecular weight higher than 250 Da. Briefly, the MR samples were incubated with a radical initiator 2,2′-azobis(2-methylpropionamidine) dihydro-chloride (AAPH), that activated the oxidation of all potential antioxidants. In a 2 mL volumetric flask, 200 µL of MR sample was added to 54 mg of AAPH radical initiator in phosphate-buffer solution (PBS) of 0.1 M at a pH of 7.4 to obtain a final concentration of ~100 mM of AAPH. The mixture was then separated into two 1.5 mL vials and incubated at 37 °C for 1 and 2 h. At 37 °C, the radical initiator starts the oxidation process, and the potential antioxidant compounds are oxidised. This method is based on the previously published study [39], that demonstrated that the peak areas of compounds with potential antioxidant activity in HPLC chromatograms are significantly reduced or disappear after incubation with AAPH, that can release ROO· at 37 °C. Once cooled in ice, the samples were analysed using HPLC coupled with HRMS, and all the spectra were processed using Compound discover software 3.3.3.200 (Thermo Fischer Scientific, Waltham, MA, USA).

### 2.6. Statistical Analyses

All statistical analyses were carried out using Compound Discoverer software (version 3.3.3.200, Thermo Fischer Scientific, Waltham, MA, USA), Microsoft Excel (version 2410, Microsoft, Redmond, WA USA) and XLSTAT plug in (version 26.3.0, Lumivero, Denver, CO, USA)

## 3. Results

### 3.1. pH Control

**Figure 1:**
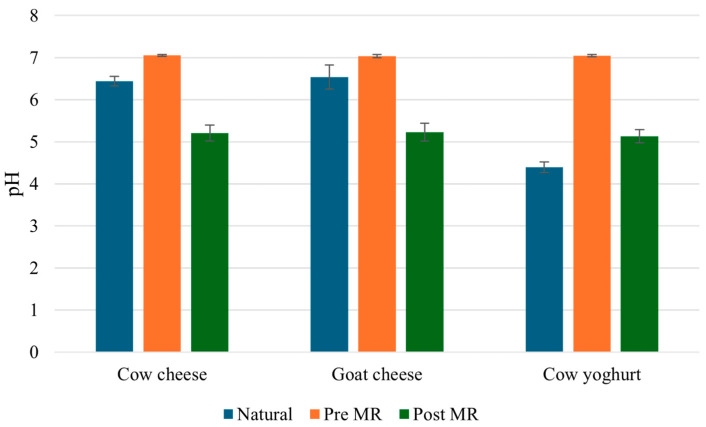
The pH measurements of natural milk wheys, after the adjustment to a pH of7 before the MR, and after the MR.

Initial pH is a key variable for the MR. If it is acidic, it decreases the velocity of the MR and favours the production of dangerous molecules such as 5-hydroxymethilfurfural, limiting the production of antioxidant MRPs [34,39]. Therefore, the pH of all samples was measured before the MR, adjusted to 7 to favour the MR, as reported in a previous work [34], and the production of antioxidant MRPs, and then measured again after MR. Values are reported in Figure 1.

Cheese wheys presented a natural pH of around 6.6, while yoghurt whey, also called acidic whey, presented a natural pH of around 4.5. All the samples, then, were brought to a pH of 7 and the MR was induced. During the Maillard Reaction, the pH naturally decreases due to the production of organic acids, such as formic and acetic acids, as products of the reaction [35]. After the MR, all the whey samples reached a similar pH of around 5, showing that the reactivity of all of samples was comparable.

### 3.2. Reactants’ Consumption

Sugars and amino acids are the main reactants of the MR. They were quantified in the three whey samples before and after the MR, induced at 140 °C for 90 min, to evaluate their presence in the starting samples and their reactivity in the MR.

#### 3.2.1. Amino Acid Consumption

**Figure 2:**
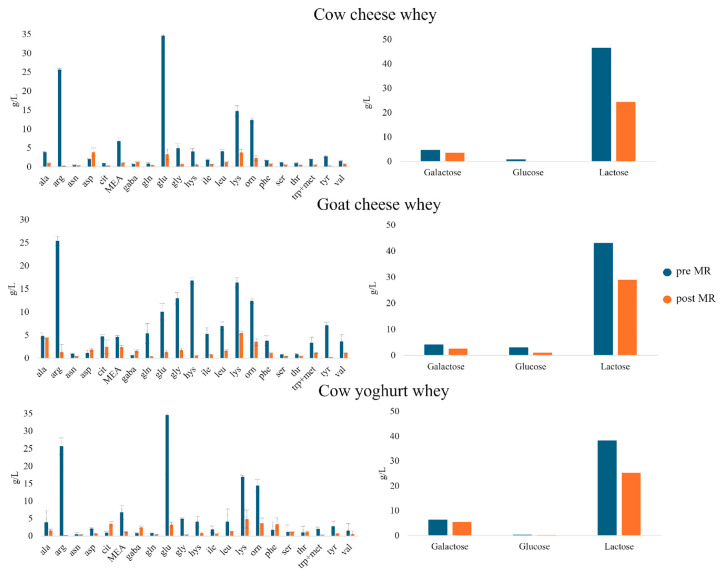
Amino acids and sugar quantification before (blu) and after (orange) Maillard reaction (MR) in samples of cow cheese whey, goat cheese whey and cow yoghurt whey. MEA = monoethanolamine.

As shown in Figure 2, the amino acidic profile of the three wheys differs mainly in the quantities of glutamic acid, glycine and histidine. In fact, cow cheese whey presents a high content of glutamic acid, like cow yoghurt whey does as well, suggesting that cow milk contains a higher quantity of this specific amino acid.

On the other hand, goat whey samples are richer in citrulline, glutamine, glycine, histidine, isoleucine, leucine, tryptophane + methionine, tyrosine and valine. All the samples, instead, presented a low content in glutamine, one of the most reactive amino acids in an MR, because of its side chain that contains an amino group, the moieties that react with reducing sugars in an MR [40]. The consumption of amino acids and sugars during the MR is a determinant in the formation of antioxidant MRPs, as these substrates provide the reactive functional groups that drive the complex cascade of chemical transformations in an MR, yielding compounds with potential antioxidant properties. Overall, from the results obtained, the amino acids can be divided into three main groups, as reported in Table 2: the most reactive (% degradation ≥ 75%), those that are medium reactive (% degradation between 50% and 75%) and those that are less reactive (% degradation ≤ 50%). The values reported are averages between the results obtained from the analyses in triplicates of every whey sample.

Moreover, it is possible to note that even if all the amino acids decreased in concentration after the MR, except for aspartic acid, GABA (γ-butyric acid) and citrulline in cow whey, arginine, that was one of the most abundant amino acids in whey and almost disappeared after the MR, showed the greatest reduction, indicating high reactivity in the MR. This result agrees with the literature, where it is reported that amino acids representing an amino group in the side chain are more reactive than others [41]. The same behaviour was followed by histidine. Lysine, instead, did not follow the same degradation pattern, resulting in its being less reactive than arginine and histidine. An interesting result, instead, is represented by the reactivity of glutamic acid where, even if it presents an acidic side chain that should limit its reactivity to a higher pH, like 8 [42], its concentration decreased almost to zero in all the samples analysed, suggesting that in the milk whey with the previously described conditions, it is reactive.

#### 3.2.2. Sugar Consumption

The sugar composition, instead, was very similar for all the whey analysed: three main sugars were present, lactose, glucose and maltose, in similar concentrations. In particular, the main sugar present was lactose, with a concentration of around 40 g/L, followed by galactose, with a concentration lower than 10 g/L and the least present was glucose, with a concentration of around 3 g/L. The low concentration of glucose may be explained by the fact that it is the favourite sugar for the fermentation of bacteria and yeasts, the organisms responsible for cheese and yoghurt production [43]. As resulted for amino acids, all the three sugars also decreased in concentration after the MR, indicating that all of them were reactive. In particular, glucose resulted in being the most reactive sugar, with ~75% consumption in goat cheese and cow cheese whey samples, while in yoghurt whey, it was present in very low concentrations. Lactose was also very reactive, with an average of consumption of ~40% in all the analysed samples. Galactose, instead, showed the least reactivity compared to the other sugars, with an average consumption of ~25% after 90 min at 140 °C. The results regarding the most reactive being glucose do agree with the literature [44], while the higher reactivity of lactose compared to galactose does not. This can be attributed to the fact that, at 140 °C, lactose undergoes hydrolysis, yielding its constituent monosaccharides, glucose and galactose, thereby increasing their concentration. Consequently, the decrease in lactose concentration is not only due to the MR but also to its hydrolysis, which additionally leads to the accumulation of the less-reactive monomer, galactose.

This interplay between substrate consumption and product formation, reported in the next chapter, highlights the fundamental role of amino acids and sugars in shaping the antioxidant potential of MRPs, setting the stage for further exploration of their specific contributions to reaction pathways.

### 3.3. Antioxidant MRPs’ Evaluation

#### 3.3.1. Samples Analyses from LAM

Using Compound Discoverer software, it was possible to analyse all the whey sampled before and after the MR, and to search specifically for the 50 *m*/*z* included in the library of antioxidant MRPs (LAM) previously created. Briefly, the LAM was built, evaluating the antioxidant potential of MR compounds produced from a mixture of 20 amino acids and 6 sugars, and assessed using HRMS and Compound Discoverer software, monitoring *m*/*z* signals that increased after the MR but decreased after incubation with a radical initiator (AAPH) [34]. To analyse the HRMS data of whey samples, an ad hoc Compound Discoverer workflow was developed. Specifically, after the alignment of the retention times of all the spectra and the integration of peaks and the detection of compounds, the data obtained were compared with the LAM based on the parent ion *m*/*z* (with a tolerance of 10 ppm), the retention time (within a 2 min tolerance), and the fragmentation profile (MS2).

With this approach, it was possible to detect in whey samples 28 *m*/*z* out of the 50 included in the LAM, and they are reported in Table 3.

In particular, it is notable that *m*/*z* n. 1, 96.0440, *m*/*z* n. 3, 110.06008, *m*/*z* n. 4, 115.03889, *m*/*z* n. 9, 127.03901 and *m*/*z* n. 14, 145.04956, that correspond to antioxidant MRPs, 2-pyrrolecarboxaldehyde, 1-methyl-2-pyrrolecarboxaldehyde, norfuraneol, maltol isomer and DDMP, respectively [34,45,46], have been detected in milk whey samples after an MR. These molecules are recognised as antioxidant compounds and contribute to the increased antioxidant activity observed during the MR in whey samples. The detection of the 28 *m*/*z* out of 50 included in the LAM demonstrates the significant production of antioxidant MRPs.

**Figure 3:**
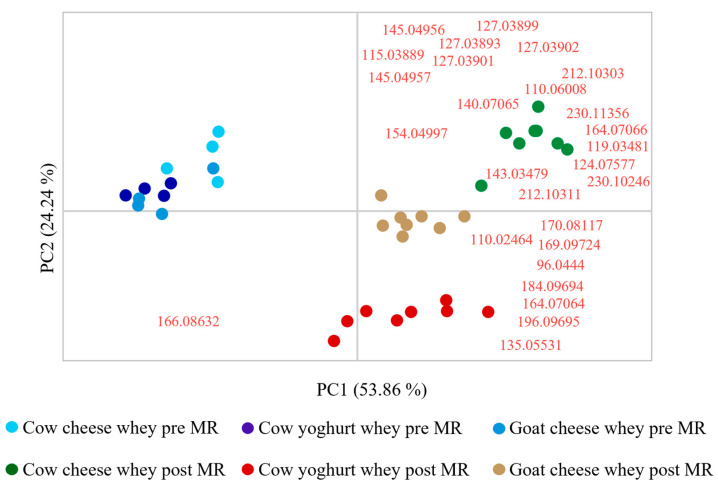
Principal component analyses (PCAs) with peak areas corresponding to *m*/*z* values included in Table 3 as variables (in red), and milk whey types before and after Maillard reaction (MR) as observations.

In Figure 3, the principal component analysis (PCA) of the data obtained is reported. The observations include all three whey samples (four samples per each whey type) analysed in duplicate before and after the MR. The variables, instead, are represented by the areas of the 28 *m*/*z* reported in Table 1 that the workflow was able to detect in whey samples.

The PCA results demonstrate a clear separation between the samples before and after the MR, as well as among whey types after the reaction. This separation underscores the diversity of MRPs formed during the process, supporting the hypothesis that starting material composition significantly influences the production of distinct antioxidant MRPs. Specifically, the clustering of post-MR samples by whey type suggests that the unique amino acid and sugar profiles of each whey contribute to generating specific MRP compositions, reinforcing the potential for tailored antioxidant compound production. In fact, the different antioxidant profiles evidenced in the PCA based on the type of whey used are crucial for future industrial applications, as they enable the selection of specific whey types based on the desired antioxidant molecules.

In particular, it is interesting to note that the known antioxidant MRPs, like 2-pyrrolecarboxaldehyde (96.0440), 1-methyl-2-pyrrolecarboxaldehyde (110.06008), maltol isomer (127.03901), DDMP (145.04956) and norfuraneol (115.03889), are mostly produced starting from cow cheese whey, and less produced by cow yoghurt whey.

#### 3.3.2. Proteins’ Reactivity

In the previous work [34], the reactivity of free amino acids was studied and used to build the LAM, but no evaluation of peptides’ and proteins’ reactivity was conducted. Studying proteins and peptides is particularly relevant because they are key reactants in the MR, yet their role in MRPs’ production remains underexplored compared to free amino acids and simple sugars. Proteins and peptides can provide a broader spectrum of amino groups and unique structural features that may influence the formation, composition, and antioxidant properties of MRPs. Understanding their contribution could reveal novel pathways and mechanisms in MRP production, offering insights into optimising these reactions for specific applications, such as food preservation or functional ingredient development. This perspective highlights the need to investigate how the complexity and diversity of proteins and peptides affect the characteristics of the resulting MRPs.

Therefore, the reactivity of peptides and proteins has been assessed, isolating the molecules with a molecular weight higher than 3 kDa and, after mixing them with reducing sugars, the MR was induced. The samples obtained were studied using the LAM previously created to see if the ions there contained were produced. In Table 4 are reported the 9 *m*/*z* out of 50 analysed that were also detected in samples where the amino compounds were just represented by molecules bigger than 3 kDa.

These findings, highlighting the marginal role of proteins in producing specific antioxidant MRPs within the LAM, are significant because they might suggest that proteins and peptides do not play a central role in MRPs’ formation and antioxidant activity. This suggests that other factors, such as the type of sugar or the presence of smaller peptides and amino acids, may be more influential in determining the antioxidant potential of MRPs. This insight also underscores the complexity of MRP chemistry and the need to further investigate how various reactants contribute to the formation of bioactive compounds.

#### 3.3.3. Bigger Molecules’ Evaluation

A limitation of the results presented here, though, is that the LAM used was obtained starting from free amino acids, and not bigger peptides or proteins. Therefore, to have a more comprehensive idea of the possible role of peptides and proteins in the production of antioxidant MRPs, the same approach based on the radical initiator reaction used to build the LAM was applied to build a second library that also includes MRPs with higher molecular weights with potential antioxidant properties. The second library includes 72 *m*/*z* values reported in Table 5 that were selected considering only peaks with areas higher than 10^6^ counts×inutes in post-MR samples and with lower areas for samples pre-MR and after the incubation with the radical initiator.

While a range of *m*/*z* values were identified, it is important to note that the antioxidant MRPs consistently did not exceed 710 *m*/*z*. This finding suggests that antioxidant activity is primarily associated with smaller, lower-molecular-weight MRPs, which could offer valuable insights into the structure–function relationships of these compounds. The presence of higher *m*/*z* values, though observed, appears to be less relevant to the antioxidant properties and may reflect unrelated or secondary reactions.

**Figure 4:**
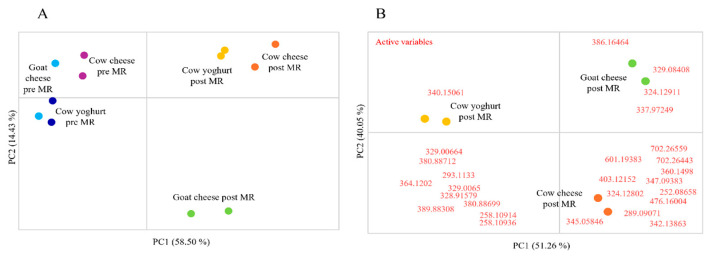
(**A**): Principal component analyses (PCA) with peak areas corresponding to *m*/*z* values included in Table 3 as variables, and milk whey type before and after Maillard reaction (MR) as observations. (**B**): PCA with peak areas corresponding to *m*/*z* values selected with partial least square discriminant analysis (PLS-DA) as variables (in red) and milk whey types after Maillard reaction (MR) as observations.

Whey samples before and after the MR were analysed considering the signal corresponding to the 72 *m*/*z* included in Table 5, and as shown in the PCA reported in Figure 4A, distinct patterns in the MRP profiles are revealed, highlighting how variations in experimental conditions influence the formation of antioxidant compounds. In particular, a separation between cow whey and goat whey is evident, and this could be related to the fact that the protein and peptides composition of the two starting milks is different. By identifying clusters that correspond to specific conditions, these findings suggest that targeted adjustments in factors such as temperature, pH, or precursor concentrations could be used to optimise the production of antioxidant MRPs. This insight opens the door for ways for refining production processes to maximise antioxidant yield, enhancing the potential application of MRPs as natural preservatives or functional ingredients. By focusing on the conditions that most significantly impact antioxidant MRP formation, future process designs can be fine-tuned to increase both the quantity and quality of these beneficial compounds.

These results suggest that the potential antioxidant MRPs detected in this study are primarily derived from proteins and peptides, rather than just free amino acids. This highlights the distinct and significant roles that both proteins/peptides and amino acids play in the production of potential antioxidant MRPs.

Applying a partial leas square discriminant analysis (PLS-DA) to only the MR data used to build the PCA in Figure 4A, it was possible to rank the variables based on their importance in the prediction (VIP), and only the variables with a VIP value higher than 1.1 were selected, since they represent the most important variables that discriminate the analysed samples. Considering only the variables selected with the PLS-DA, it was possible to build the PCA shown in Figure 4B, that allowed the identification of the variables (ions), in red, that are more produced by the MR starting from the three different types of whey.

## 4. Conclusions

The MR plays a central role in food science, not only influencing the sensory qualities of food, but also contributing to its preservation and potential health benefits related to antioxidant properties of some MRPs. The valorisation of food by-products, such as milk whey, through the application of MR offers a sustainable approach to generate antioxidant compounds, thereby reducing food waste and increasing the value of agricultural residues. This study lays the groundwork for developing sustainable approaches to improve food preservation by leveraging the antioxidant properties of MRPs derived from dairy by-products. Moreover, it elucidates how different milk whey led to the formation of some common potential antioxidant MRPs, but also how some potential antioxidant molecules are specific to one type of whey, particularly due to differences in the starting milk (cow milk or goat milk). A potential application of the identified antioxidant MRPs could be to use them in food fortification and in the development of functional ingredients, enhancing the nutritional value and providing health benefits such as improved oxidative stress defence in products like energy bars, dairy drinks, or plant-based foods. Nevertheless, before industrial application, future studies are required to optimise reaction parameters, such as temperature and reaction time, to assess their effects on reactants, including protein denaturation or aggregation and sugar activation, and to evaluate the impact of a short storage period of whey on the stability and effectiveness of the antioxidant compounds produced. Moreover, future research should also assign a chemical structure to the most interesting potential antioxidant MRPs and explore the mechanisms and efficacy of specific MRPs under different processing conditions, paving the way for new applications in food preservation that meet consumer preferences and environmental concerns.

## Figures and Tables

**Table 1:** PAD potentials and duration with Ag/AgCl electrode as reference.

Time (s)	Potential (V vs. Ag/AgCl)	Integration
0.00	1.35	Off
0.20	1.35	On
0.40	1.35	Off
0.41	−1.15	Off
0.42	−1.15	Off
0.43	1.45	Off
0.44	1.15	Off
0.50	1.15	Off

**Table 2:** Percentage of degradation (% deg) after MR at 140 °C for 90 min for every amino acid analysed, with RSD (relative standard deviation), divided into three main groups: most reactive (% degradation ≥ 75%), medium reactive (% degradation between 50% and 75%) and less reactive (% degradation ≤ 50%).

Most Reactive			Medium Reactive			Less Reactive		
Amino Acid	% deg	RSD	Amino Acid	% deg	RSD	Amino Acid	% deg	RSD
arg	97.74	2.53	MEA	71.81	21.25	ala	47.60	4.54
tyr	88.81	12.38	gln	66.03	13.92	asn	37.15	9.35
glu	89.47	2.00	ile	72.68	11.40	asp	~0	~0
gly	88.38	5.01	leu	71.81	4.21	cit	~0	20.25
hys	87.50	8.81	lys	70.77	3.79	gaba	~0	~0
orn	75.94	5.05	val	63.97	6.16	phe	10.17	1.96
trp + met	76.70	10.19				ser	38.07	9.49
						thr	32.04	2.89

**Table 3:** The *m*/*z* from the library of antioxidant MRPs (LAM) detected in milk whey samples after an MR. (*) identified known antioxidant MRP.

N°	*m*/*z*	RT [min]	Reference Ion
1 *	96.04440	3.315	[M+H]+1
2	110.02464	4.805	[M−H]−1
3 *	110.06008	5.773	[M+H]+1
4 *	115.03889	7.352	[M+H]+1
5	119.03481	4.141	[M−H] −1
6	124.07577	4.501	[M+H]+1
7	127.03893	3.564	[M+H]+1
8	127.03899	7.592	[M+H]+1
9 *	127.03901	9.546	[M+H]+1
10	127.03902	4.825	[M+H]+1
11	135.05531	6.354	[M+H]+1
12	140.07065	3.668	[M+H]+1
13	143.03479	4.822	[M−H]−1
14 *	145.04956	6.968	[M+H]+1
15	145.04957	5.683	[M+H]+1
16	154.04997	3.802	[M+H]+1
17	164.07064	4.228	[M+H]+1
18	164.07066	4.733	[M+H]+1
19	166.08632	7.866	[M+H]+1
20	169.09724	8.41	[M+H]+1
21	170.08117	3.381	[M+H]+1
22	184.09694	5.415	[M+H]+1
23	196.09695	4.183	[M+H]+1
24	212.10303	3.173	[M+H]+1
25	212.10311	6.052	[M+H]+1
26	230.10246	5.419	[M+H]+1
27	230.11343	3.486	[M+H]+1
28	230.11356	3.787	[M+H]+1

**Table 4:** The *m*/*z* from the library of antioxidant MRPs (LAM) detected in MR samples from big peptides and proteins isolates from milk whey samples. (*) identified known antioxidant MRP.

N°	*m*/*z*	RT [min]	Reference Ion
1	119.0338	3.997	[M-H]−1
2	127.0389	3.424	[M+H]+1
3	129.0546	11.096	[M+H]+1
4	140.0705	3.066	[M+H]+1
5	143.034	3.411	[M-H]−1
6	143.034	3.89	[M-H]−1
7	145.0493	6.758	[M+H]+1
8	167.0342	12.694	[M-H]−1
9	212.1028	3.055	[M+H]+1

**Table 5:** Library of *m*/*z* corresponding to MRPs with molecular weights higher than 200 Da that showed potential antioxidant activity.

Name	*m*/*z*	RT [min]	Reference Ion
Unknown 1	250.1075	5.795	[M+H]+1
Unknown 2	252.0866	5.041	[M+H]+1
Unknown 3	252.0868	5.877	[M+H]+1
Unknown 4	254.1024	6.468	[M+H]+1
Unknown 5	258.1082	3.053	[M+H]+1
Unknown 6	258.1092	3.413	[M+H]+1
Unknown 7	259.0791	4.524	[M+H]+1
Unknown 8	264.1071	3.786	[M+H]+1
Unknown 9	264.1081	4.516	[M+H]+1
Unknown 10	266.1021	5.363	[M+H]+1
Unknown 11	270.0969	4.186	[M+H]+1
Unknown 12	271.081	5.197	[M+H]+1
Unknown 13	276.1181	3.448	[M+H]+1
Unknown 14	282.1541	4.132	[M+H]+1
Unknown 15	282.1547	4.547	[M+H]+1
Unknown 16	284.1129	5.305	[M+H]+1
Unknown 17	286.09	4.504	[M+H]+1
Unknown 18	286.1393	3.108	[M+H]+1
Unknown 19	287.889	4.518	[M+H]+1
Unknown 20	289.0908	3.496	[M+H]+1
Unknown 21	293.1129	4.341	[M+H]+1
Unknown 22	293.1134	17.208	[M+H]+1
Unknown 23	299.9129	0.077	[M+H]+1
Unknown 24	301.0896	4.475	[M+H]+1
Unknown 25	304.8916	0.055	[M+H]+1
Unknown 26	305.9672	5.339	[M+H]+1
Unknown 27	306.1545	5.351	[M+H]+1
Unknown 28	324.128	2.777	[M+H]+1
Unknown 29	324.1283	3.17	[M+H]+1
Unknown 30	324.1291	5.116	[M+H]+1
Unknown 31	328.9158	4.727	[M+H]+1
Unknown 32	329.0065	5.357	[M+H]+1
Unknown 33	329.0841	5.232	[M+H]+1
Unknown 34	329.0841	4.463	[M+H]+1
Unknown 35	329.0843	5.95	[M+H]+1
Unknown 36	332.933	4.168	[M+H]+1
Unknown 37	332.9335	4.788	[M+H]+1
Unknown 38	337.9725	4.707	[M+H]+1
Unknown 39	340.15	4.117	[M+H]+1
Unknown 40	342.1385	3.509	[M+H]+1
Unknown 41	342.1386	2.777	[M+H]+1
Unknown 42	345.0583	5.248	[M+H]+1
Unknown 43	345.0585	4.861	[M+H]+1
Unknown 44	347.0938	3.655	[M+H]+1
Unknown 45	347.0942	4.076	[M+H]+1
Unknown 46	352.16	5.138	[M+H]+1
Unknown 47	352.1602	4.614	[M+H]+1
Unknown 48	360.1492	3.497	[M+H]+1
Unknown 49	360.1494	3.695	[M+H]+1
Unknown 50	364.1202	2.777	[M+H]+1
Unknown 51	375.8627	0.081	[M+H]+1
Unknown 52	380.0941	2.791	[M+H]+1
Unknown 53	380.887	0.091	[M+H]+1
Unknown 54	380.8871	4.735	[M+H]+1
Unknown 55	381.078	3.322	[M+H]+1
Unknown 56	385.9573	5.33	[M+H]+1
Unknown 57	386.1647	2.792	[M+H]+1
Unknown 58	388.1798	3.469	[M+H]+1
Unknown 59	389.8831	4.194	[M+H]+1
Unknown 60	403.1216	4.566	[M+H]+1
Unknown 61	404.8163	0.062	[M+H]+1
Unknown 62	417.8467	2.555	[M+H]+1
Unknown 63	438.1713	2.856	[M+H]+1
Unknown 64	443.047	3.28	[M+H]+1
Unknown 65	476.16	3.323	[M+H]+1
Unknown 66	476.1606	3.749	[M+H]+1
Unknown 67	487.1645	3.449	[M+H]+1
Unknown 68	601.1938	3.898	[M+H]+1
Unknown 69	649.2166	3.453	[M+H]+1
Unknown 70	689.209	3.427	[M+H]+1
Unknown 71	702.2646	3.49	[M+H]+1
Unknown 72	707.2195	3.392	[M+H]+1

## Data Availability

The authors confirm that the original contributions presented in the study are included in the article; further inquiries can be directed to the corresponding author/s.
